# HIV as a Cause of Immune Activation and Immunosenescence

**DOI:** 10.1155/2017/6825493

**Published:** 2017-10-25

**Authors:** T. Sokoya, H. C. Steel, M. Nieuwoudt, T. M. Rossouw

**Affiliations:** ^1^Department of Immunology, Faculty of Health Sciences, Institute for Cellular and Molecular Medicine, University of Pretoria, Pretoria 0001, South Africa; ^2^South African Department of Science and Technology (DST)/National Research Foundation (NRF) Centre of Excellence in Epidemiological Modelling and Analysis (SACEMA), Stellenbosch University, Stellenbosch 7600, South Africa

## Abstract

Systemic immune activation has emerged as an essential component of the immunopathogenesis of HIV. It not only leads to faster disease progression, but also to accelerated decline of overall immune competence. HIV-associated immune activation is characterized by an increase in proinflammatory mediators, dysfunctional T regulatory cells, and a pattern of T-cell-senescent phenotypes similar to those seen in the elderly. These changes predispose HIV-infected persons to comorbid conditions that have been linked to immunosenescence and inflamm-ageing, such as atherosclerosis and cardiovascular disease, neurodegeneration, and cancer. In the antiretroviral treatment era, development of such non-AIDS-defining, age-related comorbidities is a major cause of morbidity and mortality. Treatment strategies aimed at curtailing persistent immune activation and inflammation may help prevent the development of these conditions. At present, the most effective strategy appears to be early antiretroviral treatment initiation. No other treatment interventions have been found effective in large-scale clinical trials, and no adjunctive treatment is currently recommended in international HIV treatment guidelines. This article reviews the role of systemic immune activation in the immunopathogenesis of HIV infection, its causes and the clinical implications linked to immunosenescence in adults, and the therapeutic interventions that have been investigated.

## 1. Introduction

More than 3 decades following the discovery that the human immunodeficiency virus (HIV) causes the acquired immune deficiency syndrome (AIDS), there is an increasing evidence that systemic immune activation plays a significant role in the disease pathogenesis [1]. High levels of systemic immune activation and inflammation not only promote viral replication and CD4^+^ T-cell apoptosis, but also may lead to more rapid decline of immune function and competence. This resembles the phenomenon of immunosenescence that has been associated with ageing [2]. While combination antiretroviral therapy (cART) has improved the quality of life and reduced mortality and morbidity in HIV-infected persons, long-term virally suppressive treatment has not been successful in normalizing elevated markers of systemic immune activation [3]. HIV-infected individuals remain at a high risk of developing degenerative, dysfunctional, or malignant non-AIDS-defining diseases; many of which have been linked to immunosenescence and inflamm-ageing [4].

An ageing immune profile is characterized by decreased production of naïve T-cells and an increase in the proportion of memory T-cells with oligoclonal expansion [5]. The senescent T-cell phenotype is marked by a lack of CD28 expression, decreased homing receptors (e.g., CD62L and CCR7), and increased expression of the senescence marker, CD57 [6]. In addition, senescent cells are characterized by decreased proliferative capacity as indicated by shortened telomere length (TL), cell cycle arrest, increased *β*-galactosidase activity, limited proliferation in response to antigen stimulation, and activation of proinflammatory secretory pathways [6]. Several immunological changes seen in HIV-1-infected people are comparable to those observed in the aged. Proinflammatory cytokines, which are increased in HIV infection, including tumour necrosis factor (TNF)-*α*, interleukin (IL)-1*β*, and IL-6, are known to play a role in ageing [7, 8]. Increased secretion of interferon (IFN)-*α* and reduced production of IL-2 are observed in both HIV infection and ageing [9]. Similarities in T-cell differentiation also exist, such as a reduction in the longevity of CD4^+^ and CD8^+^ T-cells, reduced production of naïve CD4^+^ T-cells, increased numbers of late-differentiated CD4^+^ and CD8^+^ T-cells, and shortened TL [9].

In HIV-infected persons, systemic immune activation and CD4^+^ T-cell function are inextricably linked to immunosenescence, in what appears to be a self-perpetuating cycle. The changes in immune and cytokine release resulting from HIV-induced immune activation increase susceptibility to activation-induced cell death [10–13]; consequent immune exhaustion results in senescence and programmed CD4^+^ T-cell death, which further drive immune activation [14–17]. In both the aged and in HIV, immunosenescence has been associated with negative immune outcomes, such as thymic involution, reduction in the overall T-cell repertoire, autoimmunity, and poor antigen responsiveness [6]. Immunosenescence seems to be of particular importance in the pathogenesis of conditions where inflammation represents a significant risk factor, such as atherosclerosis and cardiovascular disease (CVD), neurodegeneration, and cancer [6]. Indeed, in the ART era, development of non-AIDS-defining, age-related comorbidities, such as osteoporosis, atherosclerosis, and neurocognitive decline, is a major cause of morbidity and mortality in HIV-infected persons [18]. The Strategies for Management of Antiretroviral Therapy (SMART) study demonstrated that deaths were mostly due to non-AIDS-defining malignancies (19%) and CVD (13%), while opportunistic diseases only accounted for 8% [19].

This study reviews the role of systemic immune activation in the immunopathogenesis of HIV infection and the causes of systemic immune activation and inflammation. We also review the clinical implications of accelerated ageing and age-related morbidity in adults and therapeutic interventions investigated to date. Data for this review were identified through searches of publicly available databases, for example, Medline and Pubmed, and in the references of studies found through these searches. Particular attention was paid to biologically *mechanistic* studies and review articles focused on systemic immune activation in HIV-infected persons. Preference was given to recent studies, that is, published in the last decade, but earlier studies that were relevant were also included.

## 2. Systemic Immune Activation in the Immunopathogenesis of HIV Infection

Introduction of HIV into host cells activates a complex network of protective responses originating from both the innate and adaptive immune systems [20]. These responses are either insufficient or too late to eliminate the virus. This enables life-long viral latency and chronic infection, which drives ongoing immune activation and progressive immunodeficiency, characterized by high cell turnover, apoptosis, and activation-induced death of immune cells [21].

Studies of pathogenic and nonpathogenic models of simian immunodeficiency virus (SIV) infection have provided insights into the role of systemic immune activation in the progression to AIDS [22]. The natural hosts of SIV, such as the African green monkey and sooty mangabey, are able to live normally with the virus and do not progress to immunodeficiency, regardless of high levels of viral replication. On the other hand, inoculating other nonhuman primates, such as rhesus macaques and Asian pigtailed macaques, with SIV results in immunodeficiency and progression to AIDS similar to that in HIV-infected humans [23–26]. During both pathogenic SIV (pSIV) and nonpathogenic SIV (npSIV) infection, robust viral replication and early antiviral responses occur during the acute phase of infection. However, it appears that the natural hosts have devised an evolutionary strategy to maintain an effective response, which enables symbiotic coexistence [27, 28]. This adaptive response appears to be associated with early resolution of acute T-cell activation, rather than an improved viral control.

It is thought that differences in immune response determine whether pSIV or npSIV infection develops. pSIV studies have demonstrated substantial loss of mucosal T-helper (Th) 17 cells, with subsequent microbial translocation as evidenced by high levels of plasma lipopolysaccharide (LPS) and soluble CD14 (sCD14) [28]. pSIV is associated with dysregulation of cell cycle and T regulatory cell (Treg) loss. This indicates a failure to the control of T-cell activation/proliferation and contributes to poor outcome [28]. Other characteristics distinguishing natural from unnatural hosts include superior cell homeostasis, higher numbers of CD4^+^ T-cells, the presence of anti-inflammatory mechanisms such as attenuated IFN signalling, maintenance of progenitor cell regeneration, and more limited immune activation, and T-cell apoptosis [27, 28].

In humans, elite controllers are a unique yet heterogeneous group of people that maintain adequate control of viral replication even in the absence of cART [22, 29]. Unlike in npSIV, elite controllers are able to downregulate viral replication in lymphoid tissue. They also have powerful and durable anti-HIV immune responses, with significantly higher activation of T-cells compared to uninfected individuals. However, this is relatively less than that seen in HIV-infected persons who are not elite controllers [29, 30]. Many elite controllers do eventually experience immune-mediated depletion of CD4^+^ T-cells and develop AIDS-defining diseases. It has been shown that basal levels of immune activation determine this progression [31].

## 3. Causes of Systemic Immune Activation in HIV

### 3.1. Direct Effects of Virions and/or Viral Proteins

HIV gene products, such as gp120 and Nef, directly stimulate the activation of lymphocytes and macrophages, resulting in the secretion of proinflammatory cytokines and chemokines [32]. Certain HIV proteins imitate and/or enhance TNF-receptor signalling, causing persistent HIV replication in infected cells through activation of nuclear factor (NF)-*κ*B, a prototypical proinflammatory signalling pathway [33], and apoptosis of uninfected bystander T-cells [34].

### 3.2. Viral Coinfections

Coinfection with other viruses, such as cytomegalovirus (CMV), Epstein-Barr virus (EBV), and hepatitis B virus (HBV) and hepatitis C virus (HCV), is common in HIV-infected individuals. Pathogenic gene products enhance the replication of HIV by transactivation of HIV long terminal repeats (LTRs) [35]. HIV-induced immunodeficiency and replicative senescence, which result in the loss of CD8^+^ T-cell populations that control viral replication, may, in turn, reactivate other pre-existing viruses or exacerbate infection by increasing viral load (VL) and consequent viral persistence [2]. This accelerates disease progression and contributes to systemic immune activation [36, 37]. CMV accounts for approximately 10% of the circulating memory T-cell repertoire in healthy, asymptomatic, HIV-uninfected CMV-seropositive individuals. The vast majority of HIV-infected individuals, between 75% and 90%, elicit significant CMV-specific T-cell responses [37, 38]. Chronic coinfection with CMV has been associated with immunological senescence, that is, gradual age-related deterioration of the immune system, homeostatic changes, and low CD4^+^ T-cell counts. It is noteworthy that the latter is particular for naïve T-cell counts, possibly due to decreased T-cell renewal capacity and thymic involution, which lead to inadequate T-cell reconstitution [39].

HIV-1-infected individuals normally have a higher content of EBV in their lymphoid tissues, or a larger number of EBV-infected cells in their peripheral blood mononuclear cells (PBMCs), than HIV-uninfected individuals. It is thought that the expansion of EBV-positive B-cells may be caused by chronic B-cell stimulation driven by HIV proteins and impaired immune surveillance against EBV secondary to immunodeficiency [40]. A strong association has been found between HIV viremia, markers of immune activation, and EBV DNA load in PBMCs [41].

Hepatocytes and Kupffer cells, the latter of which are liver macrophages, are derived from blood monocytes, phagocytose, and clear particles draining through the portal system. Decreased Kupffer and CD4^+^ T-cell counts have been found in individuals coinfected with HIV and HCV [42–44]. This cell loss may be due to the direct cytotoxic effects of HIV, specifically induced programmed cell death due to soluble viral or host factors, and altered Kupffer cell trafficking to target sites [44]. In coinfected people, elevated levels of sCD14 and LPS are found in the blood, due to a decrease in the clearance of particles and microbial products following diminished Kupffer cell numbers [42–44]. The reduction in CD4^+^ T-cells occurring during HIV-1 infection may also lead to inadequate viral control, thereby permitting reactivation of HCV, which perpetuates the cycle of viral replication and immune activation [32].

### 3.3. Persistent Elevation of Type I and II Interferons (IFNs)

IFNs I and II are produced by the innate immune system during HIV infection. IFN I plays an important role in mediating continuous inflammation. It is produced by plasmacytoid dendritic cells (pDCs) following direct activation of toll-like receptor (TLR)-7 and toll-like receptor (TLR)-8 by HIV RNA [45–47]. IFN I levels increase with plasma HIV-1 RNA levels and decrease with CD4^+^ T-cell counts [48]. IFN I leads to the synthesis and recruitment of more target cells for HIV by upregulating the HIV coreceptor, C-C chemokine receptor type 5 (CCR5), and inducing pDC production of CCR5 ligands. IFN I also suppresses thymic output, limits CD4^+^ T-cell recovery, induces CD4^+^ T-cell apoptosis, and limits antigen-specific CD4^+^ and CD8^+^ T-cell responses [49]. IFN I further stimulates expression of the immunosuppressive enzyme, indoleamine 2,3-dioxygenase (IDO), leading to dysfunctional and immunosuppressive Tregs [48]. The elevated production of IFN-*α* leads to upregulation of proapoptotic molecules [50]. The administration of IFN II to HIV-infected individuals reduces the number of CD4^+^ T-cells [49]. There is a close association between the elevation of types I and II IFN, IL-12, monocyte- and DC-derived inflammatory cytokines, and T-cell activation in HIV-infected individuals on ART [51]. The inadequate regulation of IFN responses drives chronic immune activation [52, 53].

### 3.4. Microbial Translocation

In the early stages of infection, HIV preferentially infects and depletes CCR5-expressing CD4^+^ T-cells in the gastrointestinal tract (GIT) [54–58]. The accumulation of inflammatory cells, such as pDCs, neutrophils, and monocytes, and a concomitant decrease in cells that regulate epithelial homeostasis, such as IL-17 and IL-22-producing CD4^+^ T-cells, progressively compromise mucosal integrity [59–64]. In turn, this inflammatory environment may lead to alterations in tight junction protein expression, decreased expression of claudins, upregulation of channel-forming claudins (e.g., claudin 2), and possibly increased epithelial and enterocyte apoptosis [65–69]. Dysfunction of the epithelial barrier in the GIT then allows translocation of microbial products from the intestinal lumen into the systemic circulation [70].

Pattern recognition receptors, such as nucleotide-binding oligomerization domains (NODs) and TLRs, detect microbial-associated molecular patterns (MAMPs), such as peptidoglycan, LPS, flagellin, and CpG DNA. The binding of microbial products to these receptors on cells of the innate immune system, most notably monocytes, macrophages, and DCs, activates a signalling cascade resulting in the production of proinflammatory cytokines, such as IL-1*β*, IL-6, TNF-*α*, and type-1 IFNs, such as IFN-*α* and IFN-*β* [43, 71]. For example, when TLR-4 recognises LPS, peripheral macrophages and DCs are directly stimulated to secrete proinflammatory cytokines [32]. This results in local and systemic immune activation and inflammation [65, 72–74].

Elevated levels of intestinal fatty acid-binding protein (I-FABP), originating from enterocytes, are found in the bloodstream of HIV-infected individuals [75]. I-FABP is a marker of enterocyte damage, which is associated with impaired intestinal function and microbial translocation. Enterocyte loss may be due to their reduced glucose uptake and increased expression of proinflammatory markers, such as TNF-*α* [43]. In response to the interaction between cell surface TLR-4 and monocyte activation, sCD14 is secreted into the blood [76–78]. sCD14 is a marker of LPS bioactivity and monocyte activation and is an independent predictor of mortality in HIV infection [75]. It may consequently be a clinically useful surrogate marker of immune activation [51]. The interaction between LPS and LPS binding protein (LBP) leads to activation of NF-*κ*B and increased cytokine expression. LPS-induced monocyte activation may also trigger the coagulation cascade through increased production of procoagulant tissue factor (TF), which correlates with increased levels of sCD14, D-dimer, and LBP [79]. Microbial translocation correlates with poor CD4^+^ T-cell recovery, HIV disease progression, and susceptibility to non-AIDS conditions such as CVD and neurocognitive impairment [80].

## 4. The Detrimental Consequences of Systemic Immune Activation

The detrimental consequences of systemic immune activation are multifaceted. While some are particular to HIV, for instance immune system dysregulation, many are similar to the human ageing process and affect multiple organ systems.

### 4.1. Immune System Dysregulation

Immune dysregulation is characterized by a shift in leukocyte activity and an imbalance in cytokine levels. Derangement of both the innate and adaptive immune systems is associated with increased apoptosis of CD4^+^ T- and B-cells, immunoparalysis of monocytes, and endotoxemia following microbial translocation [81]. In addition, continuous viral replication leads to a loss of T-cell homeostasis, characterized by increased T-cell turnover, an increase in the differentiation of naïve to antigen-exposed cells, T-cell replicative exhaustion, and apoptosis.

Immune activation also leads to depletion of T-cells and memory T-cell pools by other mechanisms, such as a decrease in the overall half-lives of CD4^+^ and CD8^+^ T-cells, irregular T-cell trafficking within T-cell subsets, and selective T-cell clonal exhaustion [21, 57]. A reduction in CD4^+^ T-cells compromises the host's ability to combat pathogens and results in frequent and recurrent opportunistic and nonopportunistic infections. Inhibition of the normal functions of B-cells, NK, and other antigen-presenting cells also results in suboptimal viral control, further contributing to continuous activation of the immune system [82]. T-cells reach a state of persistent replicative senescence and immune exhaustion with the loss of antigen specificity in the immune system [83].

Cytokines play a vital role in coordinating host inflammatory response and are consequently useful markers of inflammation and systemic immune activation. Excessive production of either proinflammatory, for example, IL-1*β*, IL-2, IL-6, IL-8, and TNF-*α*, or anti-inflammatory cytokines, for example, IL-4, IL-10, and IL-13, imbalances immune responses [84]. Activation of T-, B-, and NK cells by HIV antigens and their components may increase the secretion of proinflammatory cytokines, chemotactic cytokines, for example, macrophage inflammatory protein (MIP)-1*α*, and adhesion molecules associated with inflammation, such as intercellular adhesion molecule (ICAM) and vascular cell adhesion molecule (VCAM) [85–87]. Activation of monocytes, pDCs, and myeloid DCs may increase secretion of CXCL9, (monokine induced by gamma interferon (MIG)), CXCL10 (IFN gamma-induced protein 10 (IP-10)), CCL2 (monocyte chemoattractant protein-1 (MCP-1)), and TNF-*α* [51]. This culminates in T-cell activation and cytokine-driven T-cell apoptosis [88]. Increased proinflammatory cytokine levels increase susceptibility to inflammation-related conditions, such as osteoporosis, arteriosclerosis, cardiovascular conditions, and cancers [32].

Infection of pDCs by HIV may also increase immunosuppressive IDO and transforming growth factor (TGF)-*β*1, which impact immune dysregulation and T-cell homeostasis. The predominant origin of TGF-*β*1 is likely to be Tregs, but platelets, macrophages of the M2 phenotype, and immunoregulatory CD8^+^ T-cells may also produce it [88]. Activation of TGF-*β*1 signalling in fibroblasts triggers increased procollagen and chitinase 3-like-1 production. This leads to collagen deposition, tissue fibrosis, and fibroblastic reticular cell network loss within the parafollicular T-cell zone of lymph nodes [89–91]. The interaction between mucosal intestinal myofibroblasts (IMFs) and LPS also leads to an increase in the soluble mediators of fibrogenesis (IL-6 and TGF-*β*1), which directly increase collagen deposition by IMFs [92]. This may contribute to the disproportionate depletion of CD4^+^ T-cells in the GIT [90]. The ratio of Th17 to Tregs remains diminished during ART [93]. Such an imbalance may drive elevated IDO production by DCs, with subsequent impaired production of IL-17 and IL-22, leading to compromised antimicrobial immunity and tissue repair at barrier surfaces, with sustained immune activation [94, 95].

### 4.2. Thymic Function Alteration

During successful HIV suppression, CD4^+^ and CD8^+^ T-cell numbers are replenished, either through de novo thymic production, or through the proliferation of existing cells. As thymic output diminishes with age, naïve cells are mainly created through the latter process [96]. HIV infection can induce thymic damage through direct infection and killing of thymocytes, apoptosis, or disruption of the thymic stromal architecture, resulting in defective thymopoiesis and apoptosis of CD4^+^ T-cells [97]. These changes mimic those induced by ageing, characterized by a decrease in the size and compartments of the thymus, and reduced thymopoiesis [5]. Thymic involution is associated with immunosenescence, with dysfunction of the immune system secondary to alterations in T-cell composition, most notably a shift from naïve to terminally differentiated cells [5, 98]. Thymic recovery may occur in some patients on ART; however, extensive thymic damage generally hampers immune reconstitution.

Systemic immune activation, independent of CD4^+^ T-cell count and HIV VL, also results in inflammatory damage to the thymus [99]. In this case, thymic dysfunction through suboptimal production of naïve T-cells and greater differentiation of naïve into effector/memory cells occurs [100]. Immune reconstitution in HIV-infected individuals has been directly associated with thymic cellularity and volume, with the efficacy of reconstitution inversely correlated with age [101–103].

### 4.3. Systemic Inflammation

The proinflammatory state is associated with the development of major degenerative diseases in the elderly [104]. In HIV-associated immune activation, there is an increase in proinflammatory mediators, TNF-*α*, IFN-*α*, IL-2, and IL-8, and dysfunctional Tregs, which lead to such an inflammatory state. HIV-infected individuals are predisposed to chronic inflammatory conditions leading to a host of progressive age-related diseases, so-called “Inflamm-ageing” [18]. This includes inflammatory bowel disease, osteoarthritis, heart disease, kidney and liver diseases, metabolic syndrome, dementia, cancer, and frailty [105, 106]. Inflammatory biomarkers, such as C-reactive protein (CRP), IL-6, and D-dimer, are elevated in HIV-infected persons compared to HIV-uninfected persons. Randomized clinical trials have demonstrated correlations between these biomarkers, disease progression, and mortality [18, 107].

### 4.4. Development of Non-AIDS-Associated Disease

The most significant consequence of systemic immune activation, especially in terms of long-term morbidity and mortality, is the development of non-AIDS-associated diseases. In fact, increased inflammatory biomarkers are predictive of the development of non-AIDS conditions, independent of CD4^+^ T-cell count and HIV VL [32]. Many of these are also associated with ageing and have been linked to immunosenescence. The most common non-AIDS conditions related to immune activation include the following.

#### 4.4.1. Cardiovascular Disease

Individuals in the chronic phase of HIV disease have a greater risk of endothelial dysfunction and subclinical atherosclerosis than HIV-uninfected persons [108]. Endothelial dysfunction is characterized by elevated levels of endothelial lesion biomarkers and endothelial cell adhesion molecules, such as ICAM-1, VCAM-1, E-selectin, P-selectin, thrombomodulin, class 1 tissue plasminogen activator, and class 1 tissue plasminogen activator inhibitor (PAI-1) [109]. When HIV infects endothelial cells, endothelial dysfunction may result from the release of cytokines by activated monocytes or directly by gp120 and transactivator of transcription (Tat) HIV proteins altering cell signalling pathways [110, 111].

Both HIV and its treatment have been associated with vasculopathy and hypercoaguability with subsequent thrombosis [112]. *In vitro* studies have demonstrated that HIV may affect the storage and secretion of proteins that affect homeostasis, such as von Willebrand factor. HIV may also affect the fibrinolytic system through the release of TNF-*α*, which in turn increases the expression of PAI-1 in endothelial cells, a known risk factor for thrombosis. HIV proteins, specifically gp120, activate arterial smooth muscle cells to release TF, triggering coagulation through the extrinsic pathway. Conversely, HIV infection is also associated with reduced levels of anticoagulant proteins C and S and antithrombin III [113]. Thrombosis, often in the context of the metabolic syndrome, has also been linked to the protease inhibitor (PI) class of HIV treatment [114]. High levels of TNF-*α* and PAI-1, and increased expression of the scavenger receptor, CD36, with subsequent increased absorption of cholesterol, have been found in PI-treated individuals [115, 116].

A key component of atherogenesis in both HIV and ageing is the formation of lipid-laden macrophages (i.e., foam cells), secondary to unregulated uptake of modified lipoproteins, especially oxidized low-density lipoprotein (oxLDL), under the influence of CD36 [117]. HIV-infected persons have been shown to have increased levels of oxLDL and higher expression of CD36 and TLR-4 in monocytes [118]. OxLDL levels correlate with markers of monocyte activation, for example, sCD14, and TF expression on inflammatory monocytes [118]. Oxidized lipids are thought to play a role in atherosclerosis through alteration of nitric oxide (NO) signalling, initiation of endothelial activation, and expression of adhesion molecules that promote leukocyte homing [119]. The ensuing inflammatory process releases downstream biomarkers, such as IL-6, VCAM-1, selectins, fibrinogen, D-dimer, CRP, and TF, that predispose the patient to accelerated coronary atherosclerosis and arteriosclerosis and subsequent CVD including myocardial infarction, heart failure, stroke, and sudden cardiac death [120–123]. A recent mouse model has shown that the pathological process is driven by macrophages in the subendothelial space expressing senescence markers, namely elevated senescence-associated *β*-galactosidase activity, p16^Ink4a^, p53, and p21. This increases expression of key atherogenic and inflammatory cytokines and chemokines and promotes plaque instability by elevating metalloprotease production [124].

#### 4.4.2. Renal Disease

Individuals living with HIV are at an increased risk of renal diseases such as acute tubular necrosis, HIV-associated nephropathy (HIVAN) [125], HIV immune complex kidney disease, hypertensive and atherosclerotic renal diseases, and toxicity secondary to potentially nephrotoxic medication, such as tenofovir disoproxil fumarate (TDF) [126]. HIVAN is one of the major risk factors of end-stage renal disease and is histologically defined as a collapsing form of focal segmental glomerulosclerosis (FSGS), microcystic tubular dilation, tubointerstitial inflammation, and fibrosis [127]. FSGS is similar to atherosclerosis and involves the buildup of cholesterol, activation of monocytes, release of lipid-laden macrophages, and fibrosis, suggesting that similar inflammatory processes may be involved [128]. The pathogenesis of HIVAN is not entirely understood; however, it has been suggested that it is triggered by direct viral infection of renal epithelial cells, Nef-induced podocyte dysfunction, renal tubular epithelial cell apoptosis induced by Vpr, and upregulation of proinflammatory mediators, especially those induced by NF-*κ*B [127].

In ageing, senescent cells are important sources of inflammation and increased levels of biomarkers of inflammation, coagulation, and endothelial dysfunction, such as TNF-*α*, IL-6, MCP-1, CRP, and PAI-1, are commonly seen in this population [128]. Recruitment of T-cells into the renal tubulointerstitial compartment has been implicated in many renal inflammatory diseases and is an important mediator of tubular injury leading to progressive renal failure in HIVAN [129, 130]. Interactions between primary renal tubule epithelial cells (RTECs) and HIV-infected T-cells induce potent inflammatory gene responses. The consequent release of cytokines/chemokines from RTECs may then attract additional T-cells. Resident proximal tubular epithelial cells also participate in the inflammatory process by enhancing cytokine/chemokine communication with interstitial immune cells [131]. Activation of RTECs by infiltrating T-cells perpetuate local inflammatory responses through upregulation of proinflammatory chemokine/cytokine production mediated by soluble factors or by direct cell-to-cell contact [132]. The HIV-upregulated cytokines/chemokines in the RTECs include inflammatory cytokines CCL20, IL-6-, and the IL-8 related chemokines: CXCL1, CXCL2, CXCL3, CXCL5, CXCL6, and CXCL8 (IL-8). The receptors to these chemokines are expressed on certain populations of T-cells (reviewed in [133]) and, thus, may also be involved in promoting the mononuclear infiltration observed in HIVAN. The infiltration of HIV-infected cells into the kidney and activation of chemokines have been implicated in reduced survival of kidney allografts after transplantation, despite undetectable viremia [134] and the high prevalence of interstitial nephritis found in kidney biopsies in HIV-infected patients [135].

#### 4.4.3. Cognitive Impairment

HIV-infected individuals manifest a spectrum of cognitive, motor, and psychological dysfunctions similar to that found in ageing, ranging from asymptomatic neurocognitive impairment to HIV dementia. Following infection, HIV is believed to enter the central nervous system (CNS) in infected mature CD14^+^CD16^+^ monocytes that traffic to the CNS as part of the turnover of perivascular macrophages [136]. Once inside the CNS, the virus infects microglia and may remain dormant for an extended period of time. HIV does not directly destroy cells of the CNS in large quantities; instead, it triggers a cascade of deleterious inflammatory changes affecting cellular signalling and resulting in oxidative stress [137]. Proinflammatory cytokines may damage neurons, while high levels of reactive oxygen species (ROS) may damage DNA and RNA [138]. The HIV VL in the brain does not determine the extent of the inflammatory response. In individuals on ART, minuscule amounts of residual virus may be sufficient to maintain a self-perpetuating inflammatory response [137]. High levels of macrophage activation markers, such as sCD163, sCD14, and CCL2 in cerebrospinal fluid and blood, together with inflammatory biomarkers, such as CRP, IL-6, TNF-*α*, IP-10, and neopterin, have been implicated in the development of HIV-associated neurocognitive disorders (HAND) [139, 140]. This is similar to what has been observed in the elderly, where inflammatory markers, particularly IL-6 and CRP, have been linked to cognitive decline and an increased risk of dementia [141].

The CNS and microglial cells may potentially serve as anatomical and cellular reservoirs, respectively, where HIV-1 may persist during chronic infection despite successful cART. The persistence of HIV in the CNS and microglia may result in immune activation with consequent microglia senescence [142]. Brain imaging of HIV-1-infected patients on cART using positron emission tomography imaging and ^11^C-PK11195 as an *in vivo* marker of microglia activation reveals activation of microglia even in the absence of neurological symptoms [143]. The CSF from HIV-1 patients also contains increased levels of inflammatory cytokines including TNF-*α*, *β*2-microglobulin and neopterin, IL-1*α*, and S100*β* [144]. The latter, an intraneuronal calcium-inducing cytokine, could further contribute to neuronal degeneration [145]. Microglia have been demonstrated to undergo telomere shortening, which is a characteristic of senescence, in an animal model [146]. Emerging evidence from *in vitro* models also suggests that microglia could potentially develop a senescence-like phenotype characterized by reduced phagocytic and migratory capacities of microglia [147]. A dystrophic microglial phenotype has been observed to increase with ageing and has been detected in neuropathological conditions, such as Alzheimer's disease [148]. Although the progression and exact nature of microglial “ageing” remains unclear, activation and senescence appear to be integral parts of the process. Moreover, HIV-1 infection or bystander effects of HIV-1 infection seem to disrupt the delicate balance of cell survival, cell cycle progression, and apoptosis, which could contribute to the development of HAND [142].

#### 4.4.4. Osteoporosis

HIV-infected persons have an increased prevalence of osteoporotic fractures compared to age-matched, HIV-uninfected individuals [149]. In addition to traditional risk factors, such as smoking, alcohol, low body weight, and vitamin D deficiency, HIV-infected patients have additional risk factors brought about by the virus' direct and inflammatory effects on bone resorption [150], as well as the effects of ART, especially TDF [151]. The major inflammatory pathways that have been identified involved cytokines that have also been shown to be elevated during senescence [152]. For example, TNF-*α* increases the expression of the receptor activator of NF-*κ*B (RANKL), which accelerates osteoclastic bone resorption [150]. In addition, TNF-*α* and IL-1 inhibit osteoblast function and stimulate osteoblast apoptosis through activation of the inflammatory mediator, NO [152].

#### 4.4.5. Cancer

Due to immune deficiency, HIV-infected persons are at an increased risk of developing non-AIDS-defining malignancies, such as Hodgkin's lymphoma, head and neck, lung, liver, kidney, skin, and anal cancers [153, 154]. Factors contributing to the development of non-AIDs defining cancers include the virus itself, tobacco exposure, and possibly ART [154]. HIV may activate protooncogenes, alter the regulation of the cell cycle, inhibit tumour suppressor genes, or cause endothelial abnormalities, such as proangiogenesis signalling that may facilitate tumour growth and metastasis [154]. Other persistent viral coinfections commonly found in HIV-infected persons, such as HBV, HCV, human papillomavirus, and EBV, also play a role. Elevated levels of EBV-positive B-cells, which express latent membrane protein 1, a key viral protein in EBV-mediated transformation of B-cells, correlate with an increased long-term risk for such individuals to develop Hodgkin's lymphoma [40].

The risk of cancer increases with lower CD4^+^ T-cell counts; however, there appears to be an added risk even among infected people with well-preserved immune systems. CD8^+^ T-cells and NK cells maintain surveillance of the body and kill cells showing signs of anomalous growth or malignant modification. However, in HIV infection, the signalling cascades that control cell development and tissue restoration may be disrupted, leading to uncontrolled cell proliferation [155].

In HIV-infected and uninfected persons, inflammation contributes to cancer development, primarily by causing oxidative stress and DNA damage. ROS and proinflammatory cytokines, such as TNF-*α*, activate NF-*κ*B, which induces the expression of genes involved in cell proliferation, apoptosis, and carcinogenesis. This leads to further production of proinflammatory cytokines [156]. Macrophages, platelets, fibroblasts, and tumour cells are all sources of inflammatory angiogenic mediators, for example, basic fibroblast growth factor, vascular endothelial growth factor, and prostaglandin-E_1_ and E_2_ that increase the production of ROS. Additionally, many oncogenes inhibit apoptosis and, in doing so, facilitate survival of preneoplastic and malignant cells [156]. This combination of DNA damage and unchecked proliferation contribute to an increased risk of cancer.

IL-7 is important in T-cell homeostasis as it maintains the survival of the naïve T-cell pool during HIV infection [157]. Increased IL-7 leads to abnormal B-cell differentiation [158] and the upregulation of both programmed cell death protein (PD-1) and its ligands [159]. Under physiological conditions, PD-1, a negative costimulatory molecule, prevents excessive T-cell activation and assists in peripheral tolerance through promotion of Tregs [160]. The expression of PD-1, together with its cognate ligand PD-L1, is upregulated during chronic HIV infection. This is caused by the HIV Nef protein via a p38 MAPK-dependent mechanism, the cytokine-rich microenvironment, T-cell receptor-independent stimulation, and persistent activation of the innate immune system [161]. Persistently elevated levels of PD-1 expression have been observed on exhausted CD8^+^ T-cells. The PD-1/PD-L1 signalling pathway is critical in tumour immune surveillance. Tumours may escape host immune surveillance by expressing PD-L1 [162]. PD-1 signal inhibitors have emerged as a useful therapeutic strategy in the treatment of many cancers. They are also being investigated as approaches to reverse HIV latency and facilitate eradication [160, 162].

## 5. Immune Activation and Early Initiation of ART

Owing to improved ART access, the prognosis of HIV-infected patients has improved, although increased morbidity and mortality persist. This is caused by clinical events such as CVD, malignancy, and inflammatory conditions exacerbated by incomplete immune recovery and residual immune activation [29, 163]. The timing of ART initiation is thought to play an important role in immune activation [53]. Data indicate that an immunologic activation set point develops in the acute phase of HIV infection, which determines the rate at which CD4^+^ T-cells are lost over time [164]. Early ART initiation may protect and preserve lymphoid gut homeostasis and reduce microbial translocation through maintenance of epithelial integrity, maturation of mucosal DCs, and conservation of intestinal lymphoid structures [165]. Other long-term benefits include conservation of HIV-specific CD4^+^ T-cells, reduction of the turnover rate and activation of CD4^+^ and CD8^+^ T-cells, and in prevention of viral evolution [166–171].

## 6. Therapeutic Interventions

A number of therapeutic measures have been explored with the aim of reducing systemic immune activation in HIV-infected persons. To date, most studies have been observational in nature, making it impossible to rule out confounding factors, and to our knowledge, no human trials have used markers of immunosenescence as the primary outcome. Prospective interventional studies have rather focused on the causes of immunosenescence, such as immune activation and inflammation, linked with specific outcomes [6]. Unfortunately, there is no consensus regarding the optimal combination of biomarkers for measuring either immune activation or treatment success. No single strategy has been found effective in large-scale clinical trials, and no adjunctive treatment is currently recommended in international HIV treatment guidelines.

### 6.1. ART Intensification and Strengthening

Intensification with the integrase strand transfer inhibitor, raltegravir, in virally suppressed persons on ART has been found to lead to a rapid increase in 2-LTR circles with a significant decrease in levels of D-dimer [172]. Most studies have not shown any significant change in CD8^+^ T-cell activation with this strategy [173–176]. Intensification with maraviroc, a selective, reversible CCR5-receptor antagonist that inhibits the binding and signalling of CCR5 ligands, produced no effect on CD4^+^ or CD8^+^ T-cell counts and actually increased LPS and sCD14 levels [177, 178].

### 6.2. Gastrointestinal Repair Strategy

The use of prebiotics and probiotics to modify the imbalance in the bacterial profile in the GIT of HIV-infected persons has been explored. Prebiotic use showed a significant reduction in levels of sCD14 and improved the functional capability of CD4^+^ T-cells [179–181]. Supplementation with probiotics in infected macaques demonstrated reduced IDO-1 activity, indicating improved ability to maintain mucosal homeostasis [182, 183]. Other studies have shown increased CD4^+^ T-cell counts and lower levels of IL-6 and LBP with probiotic use [180, 181]. Administering bovine colostrum containing LPS-specific antibodies/immunoglobulin did not yield any significant change in LPS, sCD14 levels, or CD4^+^ T-cell counts [173, 184].

Recently, it has been reported that elite controllers, who spontaneously maintain sustained control of HIV, possess a microbiota that is richer and differs in predicted functionality from treatment naïve HIV progressors, resembling the micobiota of HIV-uninfected persons [185]. Therapeutic interventions that modulate gut microbiota richness, not only composition, are important in reducing HIV-related inflammation [185]. In addition to bacterial composition, other factors such as stability, resistance, resilience, and redundancy contribute to the functional properties of the microbiota [186]. Confirmation of microbiota-related control of HIV infection in elite controllers by metabolomic studies may result in new intervention strategies, such as faecal transplants, to control HIV [185, 187].

### 6.3. Treatment of Coinfections

Treatment of CMV seropositive patients with valganciclovir has demonstrated significant decreases in CMV DNA expression and activation of CD8^+^ T-cell, but had no effect on CRP, IL-6, and sCD14 [188]. The treatment of HCV with IFN-*α* and ribavirin did, however, correlate with a significant decrease in TNF receptor-1 and endothelial dysfunction markers, for example, soluble E-selectin and sVCAM-1 [189].

### 6.4. Interleukins

The coadministration of IL-21 and probiotics to SIV-infected animals was found to increase the production of polyfunctional Th17 and reduce pathobiont translocation [190]. Administering IL-7 to patients on ART restored functionality of CD4^+^ and CD8^+^ T-cells, enhanced CD4^+^ T-cell production, and restored intestinal Th17 and Th22 populations [191]. In addition, IL-7 significantly decreased the viral reservoir by activating latent virus replication [192]. Reconstitution of the immune system with excitatory cytokines such as IL-2 or IL-15 has improved CD4^+^ T-cell counts and HIV-specific T-cell responses [9, 193].

### 6.5. Immune Suppressive Agents

Administering cyclosporine A as a conjunctive therapy increases average CD4^+^ T-cell counts, possibly through the inhibition of T-cell activation and proliferation [194].

### 6.6. Reducing Activation of Plasmacytoid Dendritic Cells

Chloroquine and hydroxychloroquine prevent the endosomal acidification and fusion in pDCs and also inhibit IDO, a regulator of T-cell responses [195]. There is some controversy regarding the effect of chloroquine and hydroxychloroquine in HIV-infected people. Studies on chloroquine report a substantial reduction in VL in newly ART-treated patients [196, 197], a reduction in memory CD8^+^ T-cell activation and CD4^+^ and CD8^+^ T-cell proliferation [195, 198]. Additional beneficial effects, such as reduced levels of LPS, IFN-*α*, IL-6, and TNF-*α* and an increase in CD4^+^ T-cell counts, have also been demonstrated [195, 198, 199]. On the other hand, there have also been reports of no significant changes in CD4^+^ and CD8^+^ T-cell activation and proliferation [200]. An increase in VL and a reduction, or no change, in CD4^+^ T-cell counts have also been found [196, 197, 201].

### 6.7. Immune Modulators

Administering 3-hydroxy-3-methyl-glutharyl-coenzyme A (HMG-CoA) reductase inhibitors was found to reduce D-dimer and CRP [202–207]. A study of atorvastatin demonstrated a significant reduction in CD8^+^ T-cells compared to the control group [202]. Another study observed that the addition of statins to ART correlates with a decline in the occurrence of non-AIDS-associated cancer, non-Hodgkin's lymphoma, and a decreased mortality rate [206]. Selective cyclooxygenase type 2 (COX-2) inhibitors have been found to reduce CD8^+^ T-cell activation and immune activation levels [208]. The active metabolite of leflunomide, a disease-modifying antirheumatic drug, reduced activated T-cell proliferation in an *in vitro* study while no significant change was observed in HIV VL or CD4^+^ and CD8^+^ T-cell counts in patients treated with leflunomide in a randomised clinical trial [209–212]. Studies administering rapamycin and mycophenolate as a supplementary therapy with ART have shown to lower activation and proliferation of T-cells [213, 214].

### 6.8. Senolytics

Senescent cells are known to accumulate in various tissues during the aging process [215], and even a small number of these cells can cause adverse age- and disease-related phenotypes due to their “proinflammatory senescence-associated secretory phenotype” [216]. Senolytics are drugs that selectively promote apoptosis of senescent cells by temporarily disabling prosurvival signalling pathways, for example, those involving “PI3K/AKT, p53/p21/serpines, dependence receptor/tyrosine kinases, and BCL-2/BCL-X_L_.” This has delayed or alleviated the appearance of age- and disease-related phenotypes in several animal models [216]. These drugs consequently hold promise in attenuating the appearance of age-related cell phenotypes and chronic diseases, such as diabetes, pulmonary fibrosis, osteoporosis, cardiovascular disease, and cancers [216, 217].

Various drug candidates have been identified, for example, the tyrosine kinase inhibitor, dasatinib; the naturally occurring flavonoids and related compounds, such as quercetin, fisetin, and piperlongumine; drugs that target components of the BCL-2 pathway, for example, navitoclax; and the specific BCL-X_L_ inhibitors, A1331852 and A1155463 [215–219]. However, none of these drugs have demonstrated efficacy on all senescent cell types, significant side effects have been observed, none have yet successfully completed preclinical studies, and concerns exist regarding toxicity following long-term use. Fisetin, A1331852, and A1155463 appear to have more favorable side effect profiles and are potentially better candidates for use in humans [215, 216].

## 7. Conclusion

Systemic immune activation has become a focus of research into the immunopathogenesis of HIV. This immune activation is characterized by an increase in proinflammatory mediators, dysfunctional Tregs, and a pattern of T-cell-senescent phenotypes similar to those observed in the elderly. These changes predispose HIV-infected persons to comorbid conditions that have been linked to immunosenescence and inflamm-ageing. Treatment strategies aimed at curtailing persistent immune activation may help prevent the development of these conditions. At present, early ART initiation appears to be the most effective strategy although there is difficulty in achieving this in many settings. More studies of supplementary strategies are required. Consensus should also be reached regarding the optimal combination of biomarkers for measuring systemic immune activation and its successful treatment.

## References

[1] Lori F. (2008). Treating HIV/AIDS by reducing immune system activation: the paradox of immune deficiency and immune hyperactivation. *Current Opinion in HIV AIDS*.

[2] Appay V., Almeida J. R., Sauce D., Autran B., Papagno L. (2007). Accelerated immune senescence and HIV-1 infection. *Experimental Gerontology*.

[3] Hatano H. (2013). Immune activation and HIV persistence: considerations for novel therapeutic interventions. *Current Opinion in HIV and AIDS*.

[4] Sereti I., Altfeld M. (2016). Immune activation and HIV: an enduring relationship. *Current Opinion in HIV and AIDS*.

[5] Gui J., Mustachio L. M., Su D. M., Craig R. W. (2012). Thymus size and age-related thymic involution: early programming, sexual dimorphism, progenitors and stroma. *Aging and Disease*.

[6] Deeks S. G., Verdin E., McCune J. M. (2012). Immunosenescence and HIV. *Current Opinion in Immunology*.

[7] Chung H. Y., Kim H. J., Kim J. W., Yu B. P. (2001). The inflammation hypothesis of aging: molecular modulation by calorie restriction. *Annals of the New York Academy of Sciences*.

[8] Bruunsgaard H., Pedersen M., Pedersen B. K. (2001). Aging and proinflammatory cytokines. *Current Opinion in Hematology*.

[9] Appay V., Rowland-Jones S. L. (2002). Premature ageing of the immune system: the cause of AIDS?. *Trends in Immunology*.

[10] Wolthers K. C., Bea G., Wisman A. (1996). T cell telomere length in HIV-1 infection: no evidence for increased CD4^+^ T cell turnover. *Science*.

[11] Dagarag M., Ng H., Lubong R., Effros R. B., Yang O. O. (2003). Differential impairment of lytic and cytokine functions in senescent human immunodeficiency virus type 1-specific cytotoxic T lymphocytes. *Journal of Virology*.

[12] Franzese O., Adamo R., Pollicita M. (2007). Telomerase activity, hTERT expression, and phosphorylation are downregulated in CD4^+^ T lymphocytes infected with human immunodeficiency virus type 1 (HIV-1). *Journal of Medical Virology*.

[13] Meyaard L., Otto S. A., Jonker R. R., Mijnster M. J., Keet R. P., Miedema F. (1992). Programmed death of T cells in HIV-1 infection. *Science*.

[14] Aiuti F., Mezzaroma I. (2006). Failure to reconstitute CD4+ T-cells despite suppression of HIV replication under HAART. *AIDS Reviews*.

[15] Benveniste O., Flahault A., Rollot F. (2005). Mechanisms involved in the low-level regeneration of CD4^+^ cells in HIV-1-infected patients receiving highly active antiretroviral therapy who have prolonged undetectable plasma viral loads. *The Journal of Infectious Diseases*.

[16] Marchetti G., Gori A., Casabianca A. (2006). Comparative analysis of T-cell turnover and homeostatic parameters in HIV-infected patients with discordant immune-virological responses to HAART. *AIDS*.

[17] Massanella M., Negredo E., Perez-Alvarez N. (2010). CD4 T-cell hyperactivation and susceptibility to cell death determine poor CD4 T-cell recovery during suppressive HAART. *AIDS*.

[18] Nixon D. E., Landay A. L. (2010). Biomarkers of immune dysfunction in HIV. *Current Opinion in HIV and AIDS*.

[19] Strategies for Management of Antiretroviral Therapy (SMART) Study Group, El-Sadr W. M., Lundgren J. (2006). CD4^+^ count–guided interruption of antiretroviral treatment. *The New England Journal of Medicine*.

[20] Sauce D., Elbim C., Appay V. (2013). Monitoring cellular immune markers in HIV infection: from activation to exhaustion. *Current Opinion in HIV and AIDS*.

[21] Mogensen T. H., Melchjorsen J., Larsen C. S., Paludan S. R. (2010). Innate immune recognition and activation during HIV infection. *Retrovirology*.

[22] Cumont M. C., Diop O., Vaslin B. (2008). Early divergence in lymphoid tissue apoptosis between pathogenic and nonpathogenic simian immunodeficiency virus infections of nonhuman primates. *Journal of Virology*.

[23] Palmer S., Maldarelli F., Wiegand A. (2008). Low-level viremia persists for at least 7 years in patients on suppressive antiretroviral therapy. *Proceedings of the National Academy of Sciences of the United States of America*.

[24] Hatano H., Delwart E. L., Norris P. J. (2010). Evidence of persistent low-level viremia in long-term HAART-suppressed, HIV-infected individuals. *AIDS*.

[25] Brenchley J. M., Silvestri G., Douek D. C. (2010). Nonprogressive and progressive primate immunodeficiency lentivirus infections. *Immunity*.

[26] Chahroudi A., Bosinger S. E., Vanderford T. H., Paiardini M., Silvestri G. (2012). Natural SIV hosts: showing AIDS the door. *Science*.

[27] Klatt N. R., Silvestri G., Hirsch V. (2012). Nonpathogenic simian immunodeficiency virus infections. *Cold Spring Harbor Perspective in Medicine*.

[28] Lederer S., Favre D., Walters K. A. (2009). Transcriptional profiling in pathogenic and non-pathogenic SIV infections reveals significant distinctions in kinetics and tissue compartmentalization. *PLoS Pathogens*.

[29] Hunt P. W., Brenchley J., Sinclair E. (2008). Relationship between T cell activation and CD4^+^ T cell count in HIV-seropositive individuals with undetectable plasma HIV RNA levels in the absence of therapy. *The Journal of Infectious Diseases*.

[30] Saag M., Deeks S. G. (2010). How do HIV elite controllers do what they do?. *Clinical Infectious Diseases*.

[31] Noel N., Lerolle N., Lecuroux C. (2015). Immunologic and virologic progression in HIV controllers: the role of viral “blips” and immune activation in the ANRS CO21 CODEX study. *PLoS One*.

[32] Appay V., Sauce D. (2008). Immune activation and inflammation in HIV-1 infection: causes and consequences. *The Journal of Pathology*.

[33] Pasquereau S., Kumar A., Herbein G. (2017). Targeting TNF and TNF receptor pathway in HIV-1 infection: from immune activation to viral reservoirs. *Virus*.

[34] Herbein G., Khan K. A. (2008). Is HIV infection a TNF receptor signalling-driven disease?. *Trends in Immunology*.

[35] Gaardbo J. C., Hartling H. J., Gerstoft J., Nielsen S. D. (2012). Incomplete immune recovery in HIV infection: mechanisms, relevance for clinical care, and possible solutions. *Clinical and Developmental Immunology*.

[36] Operskalski E. A., Kovacs A. (2011). HIV/HCV co-infection: pathogenesis, clinical complications, treatment, and new therapeutic technologies. *Current HIV/AIDS Reports*.

[37] Hunt P. W. (2012). HIV and inflammation: mechanisms and consequences. *Current HIV/AIDS Reports*.

[38] Sylwester A. W., Mitchell B. L., Edgar J. B. (2005). Broadly targeted human cytomegalovirus-specific CD4^+^ and CD8^+^ T cells dominate the memory compartments of exposed subjects. *Journal of Experimental Medicine*.

[39] Appay V., Fastenackels S., Katlama C. (2011). Old age and anti-cytomegalovirus immunity are associated with altered T-cell reconstitution in HIV-1-infected patients. *AIDS*.

[40] Righetti E., Ballon G., Ometto L. (2002). Dynamics of Epstein–Barr virus in HIV-1-infected subjects on highly active antiretroviral therapy. *AIDS*.

[41] Petrara M. R., Cattelan A. M., Zanchetta M. (2012). Epstein-Barr virus load and immune activation in human immunodeficiency virus type 1-infected patients. *Journal of Clinical Virology*.

[42] Ancuta P., Kamat A., Kunstman K. J. (2008). Microbial translocation is associated with increased monocyte activation and dementia in AIDS patients. *PLoS One*.

[43] Sandler N. G., Douek D. C. (2012). Microbial translocation in HIV infection: causes, consequences and treatment opportunities. *Nature Reviews Microbiology*.

[44] Balagopal A., Ray S. C., De Oca R. M. (2009). Kupffer cells are depleted with HIV immunodeficiency and partially recovered with antiretroviral immune reconstitution. *AIDS*.

[45] Beignon A. S., McKenna K., Skoberne M. (2005). Endocytosis of HIV-1 activates plasmacytoid dendritic cells via toll-like receptor-viral RNA interactions. *Journal of Clinical Investigation*.

[46] Hyrcza M. D., Kovacs C., Loutfy M. (2007). Distinct transcriptional profiles in ex vivo CD4^+^ and CD8^+^ T cells are established early in human immunodeficiency virus type 1 infection and are characterized by a chronic interferon response as well as extensive transcriptional changes in CD8^+^ T cells. *Journal of Virology*.

[47] Bharaj P., Chahar H. S. (2015). Immune activation and HIV pathogenesis: implications for therapy. *Journal of Antivirals and Antiretrovirals*.

[48] Sivro A., Su R. C., Plummer F. A., Ball T. B. (2014). Interferon responses in HIV infection: from protection to disease. *AIDS Reviews*.

[49] Utay N. S., Douek D. C. (2016). Interferons and HIV infection: the good, the bad, and the ugly. *Pathogens and Immunity*.

[50] Valdez H., Lederman M. M. (1997). Cytokines and cytokine therapies in HIV infection. *AIDS Clinical Review*.

[51] Kamat A., Misra V., Cassol E. (2012). A plasma biomarker signature of immune activation in HIV patients on antiretroviral therapy. *PLoS One*.

[52] Biancotto A., Grivel J. C., Iglehart S. J. (2007). Abnormal activation and cytokine spectra in lymph nodes of people chronically infected with HIV-1. *Blood*.

[53] Rajasuriar R., Khoury G., Kamarulzaman A., French M. A., Cameron P. U., Lewin S. R. (2013). Persistent immune activation in chronic HIV infection: do any interventions work?. *AIDS*.

[54] Mehandru S., Poles M. A., Tenner-Racz K. (2004). Primary HIV-1 infection is associated with preferential depletion of CD4^+^ T lymphocytes from effector sites in the gastrointestinal tract. *Journal of Experimental Medicine*.

[55] Hayes T. L., Asmuth D. M., Critchfield J. W. (2013). Impact of highly active antiretroviral therapy initiation on CD4+ T-cell repopulation in duodenal and rectal mucosa. *AIDS*.

[56] Gordon S. N., Cervasi B., Odorizzi P. (2010). Disruption of intestinal CD4^+^ T cell homeostasis is a key marker of systemic CD4^+^ T cell activation in HIV-infected individuals. *Journal of Immunology*.

[57] Brenchley J. M., Price D. A., Schacker T. W. (2006). Microbial translocation is a cause of systemic immune activation in chronic HIV infection. *Nature Medicine*.

[58] Mavigner M., Cazabat M., Dubois M. (2012). Altered CD4^+^ T cell homing to the gut impairs mucosal immune reconstitution in treated HIV-infected individuals. *Journal of Clinical Investigation*.

[59] Klatt N. R., Estes J. D., Sun X. (2012). Loss of mucosal CD103+ DCs and IL-17+ and IL-22+ lymphocytes is associated with mucosal damage in SIV infection. *Mucosal Immunology*.

[60] Guglani L., Khader S. A. (2010). Th17 cytokines in mucosal immunity and inflammation. *Current Opinion in HIV and AIDS*.

[61] Sugimoto K., Ogawa A., Mizoguchi E. (2008). IL-22 ameliorates intestinal inflammation in a mouse model of ulcerative colitis. *Journal of Clinical Investigation*.

[62] Park H., Li Z., Yang X. O. (2005). A distinct lineage of CD4 T cells regulates tissue inflammation by producing interleukin 17. *Nature Immunology*.

[63] Brenchley J. M., Paiardini M., Knox K. S. (2008). Differential Th17 CD4 T-cell depletion in pathogenic and nonpathogenic lentiviral infections. *Blood*.

[64] Paiardini M. (2010). Th17 cells in natural SIV hosts. *Current Opinion in HIV and AIDS*.

[65] Brenchley J. M., Douek D. C. (2012). Microbial translocation across the GI tract. *Annual Review of Immunology*.

[66] Prasad S., Mingrino R., Kaukinen K. (2005). Inflammatory processes have differential effects on claudins 2, 3 and 4 in colonic epithelial cells. *Laboratory Investigation*.

[67] Chiba H., Kojima T., Osanai M., Sawada N. (2005). The significance of interferon-*γ*-triggered internalization of tight-junction proteins in inflammatory bowel disease. *Science Signaling*.

[68] Smith A. J., Schacker T. W., Reilly C. S., Haase A. T. (2010). A role for syndecan-1 and claudin-2 in microbial translocation during HIV-1 infection. *Journal of Acquired Immune Deficiency Syndromes*.

[69] Ruemmele F. M., Beaulieu J. F., Dionne S. (2002). Lipopolysaccharide modulation of normal enterocyte turnover by toll-like receptors is mediated by endogenously produced tumour necrosis factor *α*. *Gut*.

[70] Suy A., Castro P., Nomdedeu M. (2007). Immunological profile of heterosexual highly HIV-exposed uninfected individuals: predominant role of CD4 and CD8 T-cell activation. *The Journal of Infectious Diseases*.

[71] Hayashi F., Smith K. D., Ozinsky A. (2001). The innate immune response to bacterial flagellin is mediated by toll-like receptor 5. *Nature*.

[72] Shan L., Siliciano R. F. (2014). Unraveling the relationship between microbial translocation and systemic immune activation in HIV infection. *Journal of Clinical Investigation*.

[73] Klatt N. R., Harris L. D., Vinton C. L. (2010). Compromised gastrointestinal integrity in pigtail macaques is associated with increased microbial translocation, immune activation, and IL-17 production in the absence of SIV infection. *Mucosal Immunology*.

[74] Neaton J. D., Neuhaus J., Emery S. (2010). Soluble biomarkers and morbidity and mortality among people infected with HIV: summary of published reports from 1997 to 2010. *Current Opinion in HIV and AIDS*.

[75] Sandler N. G., Wand H., Roque A. (2011). Plasma levels of soluble CD14 independently predict mortality in HIV infection. *The Journal of Infectious Diseases*.

[76] Nakanjako D., Ssewanyana I., Mayanja-Kizza H. (2011). High T-cell immune activation and immune exhaustion among individuals with suboptimal CD4 recovery after 4 years of antiretroviral therapy in an African cohort. *BMC Infectious Diseases*.

[77] Fernandez S., Lim A., French M. (2009). Immune activation and the pathogenesis of HIV disease: implications for therapy. *Journal of HIV Therapy*.

[78] Ostrowski S. R. (2010). Immune activation in chronic HIV infection. *Danish Medical Bulletin*.

[79] Funderburg N. T., Mayne E., Sieg S. F. (2010). Increased tissue factor expression on circulating monocytes in chronic HIV infection: relationship to in vivo coagulation and immune activation. *Blood*.

[80] Marchetti G., Tincati C., Silvestri G. (2013). Microbial translocation in the pathogenesis of HIV infection and AIDS. *Clinical Microbiology Reviews*.

[81] Bukh A. R., Melchjorsen J., Offersen R. (2011). Endotoxemia is associated with altered innate and adaptive immune responses in untreated HIV-1 infected individuals. *PLoS One*.

[82] Muller-Trutwin M., Hosmalin A. Role for plasmacytoid dendritic cells in anti-HIV innate immunity. *Immunology and Cell Biology*.

[83] Grossman Z., Meier-Schellersheim M., Sousa A. E., Victorino R. M., Paul W. E. (2002). CD4^+^ T-cell depletion in HIV infection: are we closer to understanding the cause?. *Nature Medicine*.

[84] Duggal S., Chugh T. D., Duggal A. K. (2012). HIV and malnutrition: effects on immune system. *Clinical and Developmental Immunology*.

[85] Cohen Stuart J. W., Hazebergh M. D., Hamann D. (2000). The dominant source of CD4^+^ and CD8^+^ T-cell activation in HIV infection is antigenic stimulation. *Journal of Acquired Immune Deficiency Syndromes*.

[86] Bucy R. P., Hockett R. D., Derdeyn C. A. (1999). Initial increase in blood CD4^+^ lymphocytes after HIV antiretroviral therapy reflects redistribution from lymphoid tissues. *Journal of Clinical Investigation*.

[87] Wolf K., Tsakiris D. A., Weber R., Erb P., Battegay M. (2002). Swiss HIVCS. Antiretroviral therapy reduces markers of endothelial and coagulation activation in patients infected with human immunodeficiency virus type 1. *The Journal of Infectious Diseases*.

[88] Malherbe G., Steel H. C., Cassol S. (2014). Circulating biomarkers of immune activation distinguish viral suppression from nonsuppression in HAART-treated patients with advanced HIV-1 subtype C infection. *Mediators of Inflammation*.

[89] Estes J. D., Wietgrefe S., Schacker T. (2007). Simian immunodeficiency virus-induced lymphatic tissue fibrosis is mediated by transforming growth factor *β*1-positive regulatory T cells and begins in early infection. *The Journal of Infectious Diseases*.

[90] Estes J., Baker J. V., Brenchley J. M. (2008). Collagen deposition limits immune reconstitution in the gut. *The Journal of Infectious Diseases*.

[91] Zeng M., Smith A. J., Wietgrefe S. W. (2011). Cumulative mechanisms of lymphoid tissue fibrosis and T cell depletion in HIV-1 and SIV infections. *Journal of Clinical Investigation*.

[92] Asmuth D. M., Pinchuk I. V., Wu J. (2015). Role of intestinal myofibroblasts in HIV-associated intestinal collagen deposition and immune reconstitution following combination antiretroviral therapy. *AIDS*.

[93] Lederman M. M., Funderburg N. T., Sekaly R. P., Klatt N. R., Hunt P. W. (2013). Residual immune dysregulation syndrome in treated HIV infection. *Advances in Immunology*.

[94] Reeves R. K., Rajakumar P. A., Evans T. I. (2011). Gut inflammation and indoleamine deoxygenase inhibit IL-17 production and promote cytotoxic potential in NKp44^+^ mucosal NK cells during SIV infection. *Blood*.

[95] Favre D., Mold J., Hunt P. W. (2010). Tryptophan catabolism by indoleamine 2,3-dioxygenase 1 alters the balance of TH17 to regulatory T cells in HIV disease. *Science Translational Medicine*.

[96] Khoury G., Rajasuriar R., Cameron P. U., Lewin S. R. (2011). The role of naive T-cells in HIV-1 pathogenesis: an emerging key player. *Clinical Immunology*.

[97] Ye P., Kirschner D. E., Kourtis A. P. (2004). The thymus during HIV disease: role in pathogenesis and in immune recovery. *Current HIV Research*.

[98] Sauce D., Larsen M., Fastenackels S. (2009). Evidence of premature immune aging in patients thymectomized during early childhood. *Journal of Clinical Investigation*.

[99] Manjati T., Nkambule B., Ipp H. (2016). Immune activation is associated with decreased thymic function in asymptomatic, untreated HIV-infected individuals. *Southern African Journal of HIV Medicine*.

[100] Ho Tsong Fang R., Colantonio A. D., Uittenbogaart C. H. (2008). The role of the thymus in HIV infection: a 10 year perspective. *AIDS*.

[101] Kolte L., Dreves A. M., Ersboll A. K. (2002). Association between larger thymic size and higher thymic output in human immunodeficiency virus-infected patients receiving highly active antiretroviral therapy. *The Journal of Infectious Diseases*.

[102] Vigano A., Vella S., Principi N. (1999). Thymus volume correlates with the progression of vertical HIV infection. *AIDS*.

[103] Hakim F. T., Memon S. A., Cepeda R. (2005). Age-dependent incidence, time course, and consequences of thymic renewal in adults. *Journal of Clinical Investigation*.

[104] Howcroft T. K., Campisi J., Louis G. B. (2013). The role of inflammation in age-related disease. *Aging*.

[105] Kam L. Y., Targan S. R. (1999). Cytokine-based therapies in inflammatory bowel disease. *Current Opinion in Gastroenterology*.

[106] Olsson J., Poles M., Spetz A. L. (2000). Human immunodeficiency virus type 1 infection is associated with significant mucosal inflammation characterized by increased expression of CCR5, CXCR4, and *β*-chemokines. *The Journal of Infectious Diseases*.

[107] Boulware D. R., Hullsiek K. H., Puronen C. E. (2011). Higher levels of CRP, D-dimer, IL-6, and hyaluronic acid before initiation of antiretroviral therapy (ART) are associated with increased risk of AIDS or death. *The Journal of Infectious Diseases*.

[108] Kaplan R. C., Sinclair E., Landay A. L. (2011). T cell activation and senescence predict subclinical carotid artery disease in HIV-infected women. *The Journal of Infectious Diseases*.

[109] Macias J., Berenguer J., Japon M. A. (2009). Fast fibrosis progression between repeated liver biopsies in patients coinfected with human immunodeficiency virus/hepatitis C virus. *Hepatology*.

[110] Calza L., Pocaterra D., Pavoni M. (2009). Plasma levels of VCAM-1, ICAM-1, E-selectin, and P-selectin in 99 HIV-positive patients versus 51 HIV-negative healthy controls. *Journal of Acquired Immune Deficiency Syndromes*.

[111] Klatt N. R., Chomont N., Douek D. C., Deeks S. G. (2013). Immune activation and HIV persistence: implications for curative approaches to HIV infection. *Immunological Reviews*.

[112] Karpatkin S., Nardi M., Green D. (2002). Platelet and coagulation defects associated with HIV-1-infection. *Journal of Thrombosis and Haemostasis*.

[113] Jacobson M. C., Dezube B. J., Aboulafia D. M. (2004). Thrombotic complications in patients infected with HIV in the era of highly active antiretroviral therapy: a case series. *Clinical Infectious Diseases*.

[114] Periard D., Telenti A., Sudre P. (1999). Atherogenic dyslipidemia in HIV-infected individuals treated with protease inhibitors. The Swiss HIV cohort study. *Circulation*.

[115] Koppel K., Bratt G., Schulman S., Bylund H., Sandstrom E. (2002). Hypofibrinolytic state in HIV-1-infected patients treated with protease inhibitor-containing highly active antiretroviral therapy. *Journal of Acquired Immune Deficiency Syndromes*.

[116] Dressman J., Kincer J., Matveev S. V. (2003). HIV protease inhibitors promote atherosclerotic lesion formation independent of dyslipidemia by increasing CD36-dependent cholesteryl ester accumulation in macrophages. *Journal of Clinical Investigation*.

[117] Yu X. H., Fu Y. C., Zhang D. W., Yin K., Tang C. K. (2013). Foam cells in atherosclerosis. *Clinica Chimica Acta*.

[118] Zidar D. A., Juchnowski S., Ferrari B. (2015). Oxidized LDL levels are increased in HIV infection and may drive monocyte activation. *Journal of Acquired Immune Deficiency Syndromes*.

[119] Galkina E., Ley K. (2009). Immune and inflammatory mechanisms of atherosclerosis. *Annual Review of Immunology*.

[120] Freiberg M. S., Chang C. C., Kuller L. H. (2013). HIV infection and the risk of acute myocardial infarction. *JAMA Internal Medicine*.

[121] Butt A. A., Chang C. C., Kuller L. (2011). Risk of heart failure with human immunodeficiency virus in the absence of prior diagnosis of coronary heart disease. *Archives of Internal Medicine*.

[122] Sico J. J., Chang C. C., So-Armah K. (2015). HIV status and the risk of ischemic stroke among men. *Neurology*.

[123] Tseng Z. H., Secemsky E. A., Dowdy D. (2012). Sudden cardiac death in patients with human immunodeficiency virus infection. *Journal of the American College of Cardiology*.

[124] Childs B. G., Baker D. J., Wijshake T., Conover C. A., Campisi J., van Deursen J. M. (2016). Senescent intimal foam cells are deleterious at all stages of atherosclerosis. *Science*.

[125] Islam F. M., Wu J., Jansson J., Wilson D. P. (2012). Relative risk of renal disease among people living with HIV: a systematic review and meta-analysis. *BMC Public Health*.

[126] Meir-Shafrir K., Pollack S. (2012). Accelerated aging in HIV patients. *Rambam Maimonides Medical Journal*.

[127] Medapalli R. K., He J. C., Klotman P. E. (2011). HIV-associated nephropathy: pathogenesis. *Current Opinion in Nephrology and Hypertension*.

[128] Rifkin D. E., Sarnak M. (2009). Does inflammation fuel the fire in CKD?. *American Journal of Kidney Disease*.

[129] Becker G. J., Hewitson T. D. (2000). The role of tubulointerstitial injury in chronic renal failure. *Current Opinion in Nephrology and Hypertension*.

[130] Remuzzi G., Ruggenenti P., Benigni A. (1997). Understanding the nature of renal disease progression. *Kidney International*.

[131] van Kooten C., Daha M. R. (2001). Cytokine cross-talk between tubular epithelial cells and interstitial immunocompetent cells. *Current Opinion in Nephrology and Hypertension*.

[132] Chen P., Yi Z., Zhang W., Klotman M. E., Chen B. K. (2016). HIV infection-induced transcriptional program in renal tubular epithelial cells activates a CXCR2-driven CD4+ T-cell chemotactic response. *AIDS*.

[133] Segerer S., Nelso P. J., Schlöndorff D. (2000). Chemokines, chemokine receptors, and renal disease: from basic science to pathophysiologic and therapeutic studies. *Journal of the American Society of Nephrology*.

[134] Canaud G., Dejucq-Rainsford N., Avettand-Fenoel V. (2014). The kidney as a reservoir for HIV-1 after renal transplantation. *Journal of the American Society of Nephrology*.

[135] Parkhie S. M., Fine D. M., Lucas G. M., Atta M. G. (2010). Characteristics of patients with HIV and biopsy-proven acute interstitial nephritis. *Clinical Journal of the American Society of Nephrology*.

[136] Ivey N. S., MacLean A. G., Lackner A. A. (2009). Acquired immunodeficiency syndrome and the blood-brain barrier. *Journal of Neurovirology*.

[137] Highleyman L. (2010). Inflammation, immune activation, and HIV. *BETA*.

[138] Valdez A. N., Rubin L. H., Neigh G. N. (2016). Untangling the Gordian knot of HIV, stress, and cognitive impairment. *Neurobiology of Stress*.

[139] Bora A., Ubaida Mohien C., Chaerkady R. (2014). Identification of putative biomarkers for HIV-associated neurocognitive impairment in the CSF of HIV-infected patients under cART therapy determined by mass spectrometry. *Journal of Neurovirology*.

[140] Burdo T., Weiffenbach A., Woods S., Letendre S., Ellis R. J., Williams K. C. Elevated sCD163 is a marker of neurocognitive impairment in HIV-infected individuals on effective ART.

[141] Sartori A. C., Vance D. E., Slater L. Z., Crowe M. (2012). The impact of inflammation on cognitive function in older adults: implications for healthcare practice and research. *Journal of Neuroscience Nursing*.

[142] Chen N. C., Partridge A. T., Sell C., Torres C., Martin-Garcia J. (2017). Fate of microglia during HIV-1 infection: from activation to senescence?. *Glia*.

[143] Garvey L. J., Pavese N., Politis M. (2014). Increased microglia activation in neurologically asymptomatic HIV-infected patients receiving effective ART. *AIDS*.

[144] Tyor W. R., Glass J. D., Griffin J. W. (1992). Cytokine expression in the brain during the acquired immunodeficiency syndrome. *Annals of Neurology*.

[145] Stanley L. C., Mrak R. E., Woody R. C. (1994). Glial cytokines as neuropathogenic factors in HIV infection: pathogenic similarities to Alzheimer’s disease. *Journal of Neuropathology and Experimental Neurology*.

[146] Flanary B. E., Streit W. J. (2004). Progressive telomere shortening occurs in cultured rat microglia, but not astrocytes. *Glia*.

[147] Caldeira C., Oliveira A. F., Cunha C. (2014). Microglia change from a reactive to an age-like phenotype with the time in culture. *Frontiers of Cellular Neuroscience*.

[148] Spittau B. (2017). Aging microglia—phenotypes, functions and implications for age-related neurodegenerative diseases. *Frontiers in Aging Neuroscience*.

[149] Triant V. A., Brown T. T., Lee H., Grinspoon S. K. (2008). Fracture prevalence among human immunodeficiency virus (HIV)-infected versus non-HIV-infected patients in a large U.S. healthcare system. *Journal of Clinical Endocrinology and Metabolism*.

[150] Fakruddin J. M., Laurence J. (2003). HIV envelope gp120-mediated regulation of osteoclastogenesis via receptor activator of nuclear factor *κ*B ligand (RANKL) secretion and its modulation by certain HIV protease inhibitors through interferon-*γ*/RANKL cross-talk. *The Journal of Biological Chemistry*.

[151] Stellbrink H. J., Orkin C., Arribas J. R. (2010). Comparison of changes in bone density and turnover with abacavir-lamivudine versus tenofovir-emtricitabine in HIV-infected adults: 48-week results from the ASSERT study. *Clinical Infectious Diseases*.

[152] Ginaldi L., Di Benedetto M. C., De Martinis M. (2005). Osteoporosis, inflammation and ageing. *Immunity and Ageing*.

[153] Crum-Cianflone N. F., Hullsiek K. H., Marconi V. (2009). Trends in the incidence of cancers among HIV-infected persons and the impact of antiretroviral therapy: authors’ reply. *AIDS*.

[154] Deeken J. F., Tjen A. L. A., Rudek M. A. (2012). The rising challenge of non-AIDS-defining cancers in HIV-infected patients. *Clinical Infectious Diseases*.

[155] Peek R. M., Mohla S., DuBois R. N. (2005). Inflammation in the genesis and perpetuation of cancer: summary and recommendations from a national cancer institute-sponsored meeting. *Cancer Research*.

[156] Khansari N., Shakiba Y., Mahmoudi M. (2009). Chronic inflammation and oxidative stress as a major cause of age-related diseases and cancer. *Recent Patents on Inflammation and Allergy Drug Discovery*.

[157] Schluns K. S., Kieper W. C., Jameson S. C., Lefrancois L. (2000). Interleukin-7 mediates the homeostasis of naive and memory CD8 T cells in vivo. *Nature Immunology*.

[158] Chiodi F., Bekele Y., Lantto Graham R., Nasi A. (2017). IL-7 and CD4 T follicular helper cells in HIV-1 infection. *Frontiers in Immunology*.

[159] Kinter A. L., Godbout E. J., McNally J. P. (2008). The common *γ*-chain cytokines IL-2, IL-7, IL-15, and IL-21 induce the expression of programmed death-1 and its ligands. *Journal of Immunology*.

[160] Porichis F., Kaufmann D. E. (2012). Role of PD-1 in HIV pathogenesis and as target for therapy. *Current HIV/AIDS Reports*.

[161] Velu V., Shetty R. D., Larsson M., Shankar E. M. (2015). Role of PD-1 co-inhibitory pathway in HIV infection and potential therapeutic options. *Retrovirology*.

[162] Iwai Y., Hamanishi J., Chamoto K., Honjo T. (2017). Cancer immunotherapies targeting the PD-1 signaling pathway. *Journal of Biomedical Science*.

[163] Guaraldi G., Orlando G., Zona S. (2011). Premature age-related comorbidities among HIV-infected persons compared with the general population. *Clinical Infectious Diseases*.

[164] Deeks S. G., Kitchen C. M., Liu L. (2004). Immune activation set point during early HIV infection predicts subsequent CD4^+^ T-cell changes independent of viral load. *Blood*.

[165] Kök A., Hocqueloux L., Hocini H. (2015). Early initiation of combined antiretroviral therapy preserves immune function in the gut of HIV-infected patients. *Mucosal Immunology*.

[166] Ho D. D. (1995). Time to hit HIV, early and hard. *New England Journal of Medicine*.

[167] Rosenberg E. S., Altfeld M., Poon S. H. (2000). Immune control of HIV-1 after early treatment of acute infection. *Nature*.

[168] Mohri H., Perelson A. S., Tung K. (2001). Increased turnover of T lymphocytes in HIV-1 infection and its reduction by antiretroviral therapy. *Journal of Experimental Medicine*.

[169] Tilling R., Kinloch S., Goh L. E. (2002). Parallel decline of CD8+/CD38++ T cells and viraemia in response to quadruple highly active antiretroviral therapy in primary HIV infection. *AIDS*.

[170] Hunt P. W., Martin J. N., Sinclair E. (2003). T cell activation is associated with lower CD4^+^ T cell gains in human immunodeficiency virus-infected patients with sustained viral suppression during antiretroviral therapy. *Journal of Infectious Diseases*.

[171] Hocqueloux L., Prazuck T., Avettand-Fénoël V. (2010). Long-term immunovirologic control following antiretroviral therapy interruption in patients treated at the time of primary HIV-1 infection. *AIDS*.

[172] Hatano H., Strain M. C., Scherzer R. (2013). Increase in 2-long terminal repeat circles and decrease in D-dimer after raltegravir intensification in patients with treated HIV infection: a randomized, placebo-controlled trial. *The Journal of Infectious Diseases*.

[173] Byakwaga H., Kelly M., Purcell D. F. (2011). Intensification of antiretroviral therapy with raltegravir or addition of hyperimmune bovine colostrum in HIV-infected patients with suboptimal CD4^+^ T-cell response: a randomized controlled trial. *The Journal of Infectious Diseases*.

[174] Gandhi R. T., Zheng L., Bosch R. J. (2010). The effect of raltegravir intensification on low-level residual viremia in HIV-infected patients on antiretroviral therapy: a randomized controlled trial. *PLoS Medicine*.

[175] Hatano H., Hayes T. L., Dahl V. (2011). A randomized, controlled trial of raltegravir intensification in antiretroviral-treated, HIV-infected patients with a suboptimal CD4^+^ T cell response. *The Journal of Infectious Diseases*.

[176] Yukl S. A., Shergill A. K., McQuaid K. (2010). Effect of raltegravir-containing intensification on HIV burden and T-cell activation in multiple gut sites of HIV-positive adults on suppressive antiretroviral therapy. *AIDS*.

[177] Wilkin T. J., Lalama C. M., McKinnon J. (2012). A pilot trial of adding maraviroc to suppressive antiretroviral therapy for suboptimal CD4^+^ T-cell recovery despite sustained virologic suppression: ACTG A5256. *The Journal of Infectious Diseases*.

[178] Gutiérrez C., Diaz L., Vallejo A. (2011). Intensification of antiretroviral therapy with a CCR5 antagonist in patients with chronic HIV-1 infection: effect on T cells latently infected. *PLoS One*.

[179] Gori A., Rizzardini G., Van’t Land B. (2011). Specific prebiotics modulate gut microbiota and immune activation in HAART-naïve HIV-infected adults: results of the ‘COPA’ pilot randomized trial. *Mucosal Immunology*.

[180] Rakoff-Nahoum S., Paglino J., Eslami-Varzaneh F., Edberg S., Medzhitov R. (2004). Recognition of commensal microflora by toll-like receptors is required for intestinal homeostasis. *Cell*.

[181] Klatt N. R., Canary L. A., Sun X. (2013). Probiotic/prebiotic supplementation of antiretrovirals improves gastrointestinal immunity in SIV-infected macaques. *The Journal of Clinical Investigation*.

[182] Tincati C., Douek D. C., Marchetti G. (2016). Gut barrier structure, mucosal immunity and intestinal microbiota in the pathogenesis and treatment of HIV infection. *AIDS Research and Therapy*.

[183] Vujkovic-Cvijin I., Swainson L. A., Chu S. N. (2015). Gut-resident lactobacillus abundance associates with IDO1 inhibition and Th17 dynamics in SIV-infected macaques. *Cell Reports*.

[184] Trois L., Cardoso E. M., Miura E. (2008). Use of probiotics in HIV-infected children: a randomized double-blind controlled study. *Journal of Tropical Pediatrics*.

[185] Vesterbacka J., Rivera J., Noyan K. (2017). Richer gut microbiota with distinct metabolic profile in HIV infected elite controllers. *Scientific Reports*.

[186] Moya A., Ferrer M. (2016). Functional redundancy-induced stability of gut microbiota subjected to disturbance. *Trends in Microbiology*.

[187] Hensley-McBain T., Zevin A. S., Manuzak J. (2016). Effects of fecal microbial transplantation on microbiome and immunity in simian immunodeficiency virus-infected macaques. *Journal of Virology*.

[188] Hunt P. W., Martin J. N., Sinclair E. (2011). Valganciclovir reduces T cell activation in HIV-infected individuals with incomplete CD4^+^ T cell recovery on antiretroviral therapy. *The Journal of Infectious Diseases*.

[189] Guzmán-Fulgencio M., Berenguer J., de Castro I. F. (2011). Sustained virological response to interferon-*α* plus ribavirin decreases inflammation and endothelial dysfunction markers in HIV/HCV co-infected patients. *The Journal Antimicrobial Chemotherapy*.

[190] Ortiz A. M., Klase Z. A., SR D. N. (2015). IL-21 and probiotic therapy improve Th17 frequencies, microbial translocation, and microbiome in ARV treated, SIV-infected macaques. *Mucosal Immunology*.

[191] Paiardini M. (2011). Hijacking the IL-7/IL-7R system in HIV infection. *Journal of Leukocyte Biology*.

[192] Chomont N., El-Far M., Ancuta P. (2009). HIV reservoir size and persistence are driven by T cell survival and homeostatic proliferation. *Nature Medicine*.

[193] Wilson E. M., Sereti I. (2013). Immune restoration after antiretroviral therapy: the pitfalls of hasty or incomplete repairs. *Immunological Reviews*.

[194] Emmel E. A., Verweij C. L., Durand D. B., Higgins K. M., Lacy E., Crabtree G. R. (1989). Cyclosporin a specifically inhibits function of nuclear proteins involved in T cell activation. *Science*.

[195] Martinson J. A., Montoya C. J., Usuga X., Ronguillo R., Landay A. L., Desai S. N. (2010). Chloroquine modulates HIV-1-induced plasmacytoid dendritic cell alpha interferon: implication for T-cell activation. *Antimicrobial Agents and Chemotherapy*.

[196] Sperber K., Louie M., Kraus T. (1995). Hydroxychloroquine treatment of patients with human immunodeficiency virus type 1. *Clinical Therapeutics*.

[197] Sperber K., Chiang G., Chen H. (1997). Comparison of hydroxychloroquine with zidovudine in asymptomatic patients infected with human immunodeficiency virus type 1. *Clinical Therapeutics*.

[198] Piconi S., Parisotto S., Rizzardini G. (2011). Hydroxychloroquine drastically reduces immune activation in HIV-infected, antiretroviral therapy-treated immunologic nonresponders. *Blood*.

[199] Murray S. M., Down C. M., Boulware D. R. (2010). Reduction of immune activation with chloroquine therapy during chronic HIV infection. *Journal of Virology*.

[200] Raffatellu M., Santos R. L., Verhoeven D. E. (2008). Simian immunodeficiency virus-induced mucosal interleukin-17 deficiency promotes salmonella dissemination from the gut. *Nature Medicine*.

[201] Paton N. I., Goodall R. L., Dunn D. T. (2012). Effects of hydroxychloroquine on immune activation and disease progression among HIV-infected patients not receiving antiretroviral therapy: a randomized controlled trial. *JAMA*.

[202] Ganesan A., Crum-Cianflone N., Higgins J. (2011). High dose atorvastatin decreases cellular markers of immune activation without affecting HIV-1 RNA levels: results of a double-blind randomized placebo controlled clinical trial. *The Journal of Infectious Diseases*.

[203] De Wit S., Delforge M., Necsoi C. V., Clumeck N. (2011). Downregulation of CD38 activation markers by atorvastatin in HIV patients with undetectable viral load. *AIDS*.

[204] Aslangul E., Fellahi S., Assoumou L. K., Bastard J. P., Capeau J., Costagliola D. (2011). High-sensitivity C-reactive protein levels fall during statin therapy in HIV-infected patients receiving ritonavir-boosted protease inhibitors. *AIDS*.

[205] Fichtenbaum C. J., Yeh T. M., Evans S. R., Aberg J. A. (2010). Treatment with pravastatin and fenofibrate improves atherogenic lipid profiles but not inflammatory markers in ACTG 5087. *Journal of Clinical Lipidology*.

[206] Chao C., Xu L., Abrams D. I. (2011). HMG-CoA reductase inhibitors (statins) use and risk of non-Hodgkin lymphoma in HIV-positive persons. *AIDS*.

[207] Moore R. D., Bartlett J. G., Gallant J. E. (2011). Association between use of HMG CoA reductase inhibitors and mortality in HIV-infected patients. *PLoS One*.

[208] Pettersen F. O., Torheim E. A., Dahm A. E. (2011). An exploratory trial of cyclooxygenase type 2 inhibitor in HIV-1 infection: downregulated immune activation and improved T cell-dependent vaccine responses. *Journal of Virology*.

[209] Schlapfer E., Fischer M., Ott P., Speck R. F. (2003). Anti-HIV-1 activity of leflunomide: a comparison with mycophenolic acid and hydroxyurea. *AIDS*.

[210] Davis J. P., Cain G. A., Pitts W. J., Magolda R. L., Copeland R. A. (1996). The immunosuppressive metabolite of leflunomide is a potent inhibitor of human dihydroorotate dehydrogenase. *Biochemistry*.

[211] Cherwinski H. M., Cohn R. G., Cheung P. (1995). The immunosuppressant leflunomide inhibits lymphocyte proliferation by inhibiting pyrimidine biosynthesis. *The Journal of Pharmacologyand Experimental Therapeutics*.

[212] Read S. W., DeGrezia M., Ciccone E. J. (2010). The effect of leflunomide on cycling and activation of T-cells in HIV-1-infected participants. *PLoS One*.

[213] Chapuis A. G., Paolo Rizzardi G., D’Agostino C. (2000). Effects of mycophenolic acid on human immunodeficiency virus infection in vitro and in vivo. *Nature Medicine*.

[214] Di Benedetto F., Di Sandro S., De Ruvo N. (2010). First report on a series of HIV patients undergoing rapamycin monotherapy after liver transplantation. *Transplantation*.

[215] Kirkland J. L., Tchkonia T. (2017). Cellular senescence: a translational perspective. *eBioMedicine*.

[216] Chang J., Wang Y., Shao L. (2016). Clearance of senescent cells by ABT263 rejuvenates aged hematopoietic stem cells in mice. *Nature Medicine*.

[217] Zhu Y., Doornebal E. J., Pirtskhalava T. (2017). New agents that target senescent cells: the flavone, fisetin, and the BCL-XL inhibitors, A1331852 and A1155463. *Aging*.

[218] Schafer M. J., White T. A., Iijima K. (2017). Cellular senescence mediates fibrotic pulmonary disease. *Nature Communications*.

[219] Wang Y., Chang J., Liu X. (2016). Discovery of piperlongumine as a potential novel lead for the development of senolytic agents. *Aging*.

